# Words as Visual Objects: Neural and Behavioral Evidence for High-Level Visual Impairments in Dyslexia

**DOI:** 10.3390/brainsci11111427

**Published:** 2021-10-28

**Authors:** Heida Maria Sigurdardottir, Inga María Ólafsdóttir, Hélène Devillez

**Affiliations:** Icelandic Vision Lab, Department of Psychology, University of Iceland, Saemundargata 12, 102 Reykjavik, Iceland; ingamaria@hi.is (I.M.Ó.); hdevillez@hi.is (H.D.)

**Keywords:** dyslexia, face perception, high-level vision, object perception, reading

## Abstract

Developmental dyslexia is defined by reading impairments that are disproportionate to intelligence, motivation, and the educational opportunities considered necessary for reading. Its cause has traditionally been considered to be a phonological deficit, where people have difficulties with differentiating the sounds of spoken language. However, reading is a multidimensional skill and relies on various cognitive abilities. These may include high-level vision—the processes that support visual recognition despite innumerable image variations, such as in viewpoint, position, or size. According to our high-level visual dysfunction hypothesis, reading problems of some people with dyslexia can be a salient manifestation of a more general deficit of high-level vision. This paper provides a perspective on how such non-phonological impairments could, in some cases, cause dyslexia. To argue in favor of this hypothesis, we will discuss work on functional neuroimaging, structural imaging, electrophysiology, and behavior that provides evidence for a link between high-level visual impairment and dyslexia.

## 1. Introduction

Children and adults with developmental dyslexia have reading impairments that are disproportionate to their intelligence, motivation, and educational opportunities considered necessary for reading. Converging evidence indicates that dyslexia involves a disorder of the language system, primarily a phonological processing deficit [1,2,3,4,5,6,7]. Such difficulties have been defined as problems with the sensitivity to individual sounds of spoken language [7] and can manifest as difficulties with articulation, word retrieval, and verbal memory, to name a few examples [2].

While the phonological view dominates the field, reading is a complicated skill that must rely on several cognitive abilities, not just phonological processing. Accordingly, reading problems have been associated with a wide variety of difficulties, such as a temporal processing deficit [8,9,10], slowed visual processing [11], developmental impairments of magnocellular neurons [12], visual attentional deficits [13], and difficulties with rapid automatized naming (RAN) [14,15]. Moreover, two persons showing the same pattern of reading deficits can have very different neural responses to reading [16], and there appear to be several distinct, additive risk factors for reading disability [17]. Lastly, the influence of phonological awareness in dyslexia seems to be modulated by the orthography of languages, where it is less associated with reading outcomes in languages with shallow, or more transparent, orthographies [18]. Dyslexia is likely a heterogeneous disorder, an umbrella term for reading deficits of various causes.

In this paper, we argue for the perspective that some people with developmental dyslexia have a disorder of high-level vision. By high-level vision, we mean the visual processes that are dedicated to analyzing the structure of our surroundings, particularly recognizing objects and other things despite countless variations in viewpoint, position, size, lighting, or visual clutter [19]. Several studies on readers with dyslexia provide evidence for unusual or deficient high-level visual neural mechanisms as well as impaired performance in tasks believed to depend on high-level regions of the ventral visual stream. Here we will argue that the most parsimonious explanation for this is provided by the high-level visual dysfunction hypothesis: Reading problems in dyslexia can, for some readers with dyslexia, be a salient manifestation of a more general deficit of high-level vision. As these visual processes are assumed to be supported by higher levels of the ventral visual stream [20] (see below), we also refer to this as the ventral view of dyslexia. To argue in favor of this hypothesis, we will discuss work on functional neuroimaging, structural imaging, electrophysiology, and behavior that provides evidence of a link between high-level visual impairment and dyslexia.

## 2. The Role of Vision in Dyslexia

As early as the 19th century, what would later be called dyslexia was described as “word-blindness”, “text-blindness”, or “letter-blindness” [21,22,23,24]. Kussmaul [21] first claimed that “a complete text-blindness may exist, although the power of the sight, the intellect, and the powers of speech are intact”. Hinshelwood [22] described a man unable to read despite having normal visual acuity, a fact that Hinshelwood attributed not “to any failure of visual power, but to a loss of the visual memory of letters”. Similarly, Morgan [24] described a boy who clearly saw words, but had “no power of preserving and storing up the visual impression produced by words”. These authors all seem to have agreed that the described reading impairments were not related to low-level visual deficits, such as lower visual acuity. Such problems might, however, be attributed to deficits in visual cognition or high-level vision. The view of these early researchers nonetheless seems to have been that the impairments only applied to letters and words and would not generalize to other visual objects. Nevertheless, letters and words *are* visual objects—albeit special ones—that must be extensively processed by the visual system before they can be recognized. According to the high-level visual dysfunction hypothesis, the difficulties of readers with dyslexia are not always confined to written material but can generalize to the visual discrimination and recognition of other objects.

How can this be, one might ask? After all, readers with dyslexia clearly see the world and there should be nothing wrong with their eyes, so how can their problems be visual? This may be more than just a straw man. We often feel like we are aware of all our surroundings and that we instantly and effortlessly recognize the things in our environment. Vision feels easy. It feels so easy, in fact, that the task of building an artificial system that could essentially mimic the human visual system was famously given to a few MIT students as a summer project over half a century ago [25]. Computer vision has only recently come anywhere close to reaching this goal [26]—and no wonder; the primate visual system is incredibly complex, with several dozen interconnected visual cortical areas [27,28]. Vision is not trivial at all, and many things can go wrong from the time that light hits our eyes to the moment that we understand what we see.

## 3. The Visual System

**Figure 1:**
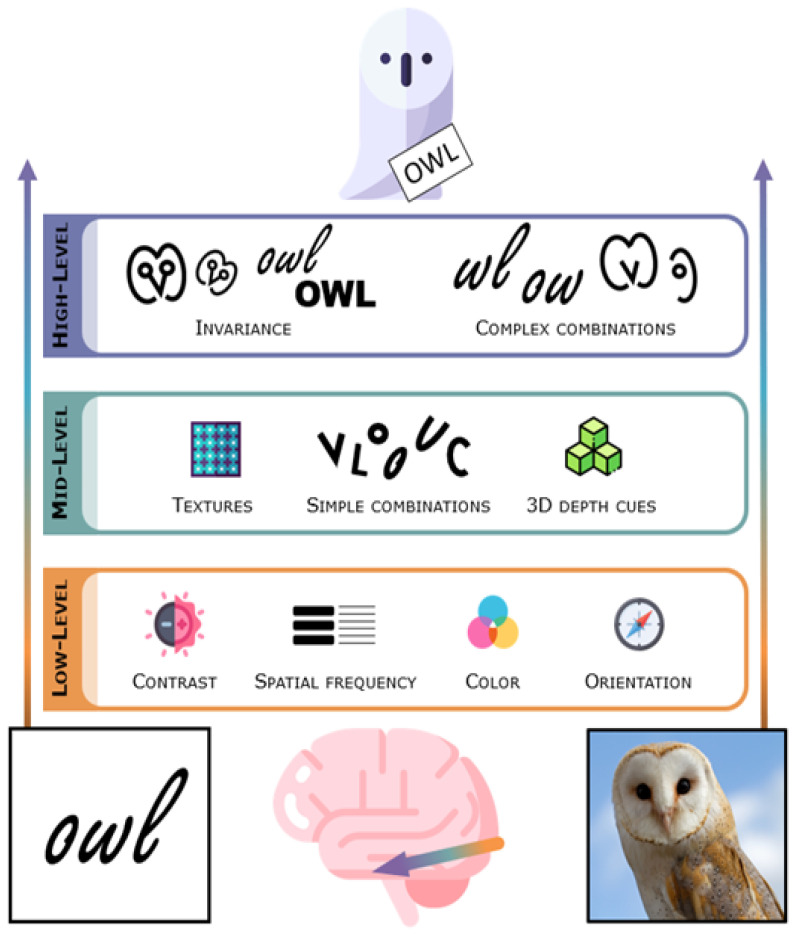
Simplified schematic of the hierarchical view of vision based on studies in humans and animals. Visually presented words are generally believed to go through the entire visual processing hierarchy [46]: low-level (contrast, spatial frequencies, color or orientation), mid-level (simple conjunctions of visual features without necessarily being driven by any of the constituent parts [47]) and high-level (combinations of mid-level features as complex shapes [43] and tolerance or invariance to identity-preserving transformations [41,48,49]). Words likely share low- and mid-level features with various objects. Words and other object classes may, however, recruit specialized high-level visual features as they are not necessarily characteristic of all object types, and this might need to be learned through experience [50]. Figure inspired by figures from Groen et al. [51] and Dehaene et al. [46], icons by the current authors or by Flaticon.com, owl picture by Tony Hisgett, under a CC BY 2.0 license: https://creativecommons.org/licenses/by/2.0/ (accessed on 20 September 2021).

Light that enters the eye gets transduced into neural signals in the retina. Retinal ganglion cells project to the subcortical lateral geniculate nucleus (LGN) of the thalamus, and these in turn project to the primary visual cortex (striate cortex, V1) in the occipital lobe in the posterior brain. Along the way to the cortex, the visual signal has already been preprocessed to accentuate features important for finding and segmenting objects [29]. The lateral geniculate nucleus and the primary visual cortex are nonetheless often thought of as low-level visual regions as they contain neurons sensitive to fundamental image characteristics such as contrast, spatial frequency (overall global changes vs. details and edges), color, and orientation of visual stimuli in confined parts of the visual field [30,31,32] (see Figure 1).

Visual cortical regions are roughly separated into the dorsal and ventral visual streams. Each stream receives neural signals mainly from the primary visual cortex, but the ventral stream proceeds to the temporal cortex while the dorsal stream progresses towards the parietal cortex. The dorsal visual stream is sensitive to the location of objects and is thought to support interaction with objects in our surroundings, such as looking at them, reaching for them, and grasping or throwing them [33,34,35,36]. It is therefore often described as the “where” or “how” pathway. While the dorsal stream almost surely plays an important role in reading, it is not the topic of this review. The ventral visual stream, often referred to as the “what” pathway, supports object identification and discrimination [33,34,35,36]. The ventral stream solves the hard problem of high-level vision; we need to recognize countless objects, and the same object can appear to us in an almost infinite number of ways, projecting completely different images onto the retina of our eyes.

**Figure 2:**
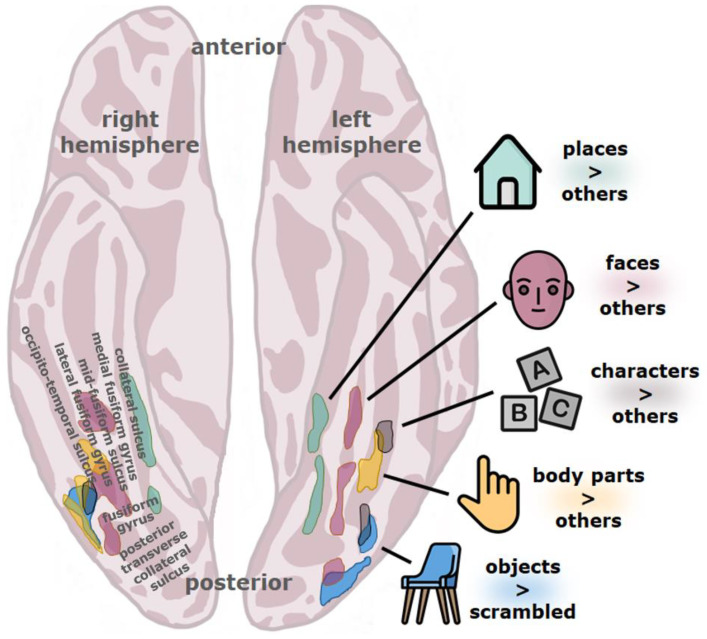
Topographical organization of the high-level ventral visual stream. Regions are defined by contrasting the activity evoked by a category with activity evoked by other stimuli, represented by >. Place-selective regions (cyan) are defined by places > (faces, characters, body parts, objects), face-selective regions (mauve) by faces > (places, characters, body parts, objects), character-selective regions (grey) by characters > (places, faces, body parts, objects), body part-selective regions (yellow) by body parts > (places, faces, characters, objects), and object-selective regions (blue) by objects > scrambled objects (which share low-level visual qualities with objects). Character-selective regions tend to be found within the occipito-temporal sulcus. They partially overlap with face-selective, body part-selective, and object-selective regions. The visual word form area (VWFA), as traditionally defined, corresponds to the posterior character-selective area of the left hemisphere. Figure adapted from Grill-Spector and Weiner [37] with author permission. Icons by Flaticon.com. Visual stimuli used to define functional regions can be found at: https://github.com/VPNL/fLoc (accessed on 20 September 2021).

The ventral stream is typically considered to be hierarchical, consisting of a number of different stages which form increasingly abstract visual representations as one goes further anterior along this pathway (see Figure 1) [33,35,36,37,38,39,40,41,42,43], although recurrence surely also plays a role [44,45]. In the human visual system, the stream originates in the primary visual cortex (V1), goes through several other retinotopically organized cortical regions (V2, V3, hV4), and extends to the ventral temporal cortex, an anatomical region that includes the fusiform gyrus (also named the lateral occipito-temporal gyrus), parahippocampal gyrus, and bounding sulci, including the occipito-temporal sulcus [37]. The ventral temporal cortex is the home of several high-level visual regions (see Figure 2) that process the visual properties of objects and serve visual perception and recognition [37]. Among these are character-selective regions that tend to respond more vigorously to visually presented text compared to images of different types of objects.

Visual words, just like all other visual objects, go through extensive processing along this cortical hierarchy [52], and high-level regions of the ventral visual stream are important for visual word recognition. Damage to the left fusiform gyrus and adjacent tissue can give rise to severe reading problems [53,54,55], and such reading problems can be transiently induced when parts of the left fusiform gyrus and the adjoining inferior temporal gyrus are deactivated. These problems are quite specific, as several other functions such as spontaneous speech, auditory comprehension, and writing are spared by stimulation of several of the implanted electrodes [56]. The neuronal recycling hypothesis [57] supposes that because of experience with reading, these regions are recycled for the purpose of recognizing written words. The recycling hypothesis furthermore assumes that such cortical plasticity is constricted by the evolutionary history of the cortex. In other words, these regions are selected for recycling because visual word recognition demands certain neural characteristics that these cortical areas happen to have [57]; see also [58,59,60]. The character-selective cortical region that has gained the greatest attention is the visual word form area (VWFA) which has been the focus of several studies on developmental dyslexia.

## 4. The Visual Word Form Area (VWFA)

The visual word form area (VWFA), as the name implies, is thought to support reading. It emerges with literacy, is recruited during reading, and perturbing its function impairs reading [55,56,57,61]. The VWFA is found in the occipito-temporal sulcus extending into the lateral fusiform gyrus, although the occipito-temporal sulcus most consistently predicts its location [37,62]. In general, the VWFA responds more to print than during rest, visual fixation, or during the visual presentation of other stimuli, such as checkerboards, consonant strings, or nonsense characters [63,64]; for a review, see e.g., [57,65,66]. The VWFA is found within or near cortical regions that mainly encode high-level visual feature classes such as irregular patterns, object parts, and entire objects [37,67]. It has also been shown that the VWFA is found within a larger region of the ventral temporal cortex that reacts more to foveally presented visual stimuli than to peripheral stimuli and that responds more to objects of small real-world size rather than large [37]. The VWFA also has a low temporal processing capacity compared to other nearby regions [68]. The VWFA might therefore respond particularly well to words because words happen to be small, static, complex visual objects most often viewed in the center of the visual field. The VWFA is also highly left-lateralized [69], although an analogous right hemisphere region can sometimes be found [37,70], and is unusually well connected to language areas [71]. Finally, the activity of the VWFA changes after particular types of visual experience that might also be important for reading, such as visual associative and visual statistical learning [72,73]. Input (visual factors), output (language factors), and the need for plasticity might thus govern the location of the VWFA. Any problems with the structure or function of such a high-level region of the ventral visual stream could lead to reading problems.

The VWFA, despite its name, also responds to many visually presented objects other than words (e.g., symbols, tools, faces), and its activity for non-words can even exceed that for words [65,74,75,76]. As an example, Starrfelt and Gerlach [77] looked at VWFA activity for both words and pictures (line drawings) while people performed various tasks. When people had to decide whether line drawings were of real or nonsense objects, a task that requires fine-grained shape analysis, VWFA activation numerically exceeded that for words. The authors suggested that the activity of the VWFA reflects the “integration of shape elements into more elaborate shape descriptions corresponding to whole objects or large object parts” [77]. The authors furthermore suggested that deciding whether or not something is a real object requires object individuation or subordinate-level categorization (e.g., canary) as opposed to basic-level (e.g., bird) or superordinate-level (e.g., animal) categorization [77]. The former requires the most detailed shape descriptions [78,79,80]. Therefore, abnormalities in the visual word form area might lead to problems with reading and predispose people to other problems with visual cognition, such as subtle deficits in tasks that involve fine-grained analysis of shape. As detailed below, people with or at a risk for dyslexia indeed show evidence for functional and possibly even structural abnormalities in the visual word form area, which might extend to other regions of the ventral visual stream.

## 5. Neural Evidence

### 5.1. Functional Neuroimaging

One of the strongest pieces of evidence for a possible role of high-level vision in dyslexia is that regions far along the ventral visual stream of dyslexic readers consistently show functional abnormalities (see e.g., [81,82]). A meta-analysis of functional imaging studies of adults and children with dyslexia performing reading-related tasks revealed consistent hypoactivation of loci within the left ventral visual stream, more specifically in the left fusiform gyrus and nearby regions [83]. This likely includes the VWFA. While other over- and underactive regions were reported in both cases, clusters of underactivation in and around the left fusiform gyrus were the only ones that overlapped in children and adults with dyslexia, pointing to their fundamental contribution to the etiology of the disorder (see also discussion in [84]).

These functional abnormalities appear to be specific to readers with dyslexia. A recent study by Banfi et al. [85] found no functional differences between children with isolated spelling deficits and typical readers, while children with dyslexia showed lower activity in several brain regions, including the left fusiform gyrus (see also [86]). Similarly, readers with dyslexia showed left occipito-temporal hypoactivation while people with specific reading comprehension deficits and intact word-level abilities did not [87].

The functionality of high-level visual regions might contribute to reading problems across languages. A left occipito-temporal hypoactivation is consistently found in dyslexic readers for both deep (e.g., English) and shallow (e.g., Italian) orthographies [88] and even for dyslexic readers of languages with a logographic script (Chinese) [89,90]. Higher convergence of hypoactive regions in the left fusiform gyrus is nonetheless found across studies for languages with shallow compared to deep orthographies [88]. It might therefore be that high-level visual factors play a larger part in reading deficits for languages with greater grapheme-phoneme correspondence.

Hypoactivity in bilateral regions of the ventral visual stream (fusiform/occipito-temporal gyri) is already present in preliterate children with a familial risk for dyslexia [91]. This appears to be restricted to those who later actually develop a reading deficit. A recent longitudinal study by Centanni et al. [92] measured fMRI activation in bilateral fusiform gyri of pre-reading children with and without risk of future dyslexia. At-risk children who later developed reading problems showed hypoactivation in the left fusiform gyrus whereas children who did not develop reading problems showed no hypoactivation, regardless of their familial risk status. As abnormalities of high-level ventral stream regions predict later reading problems, they are unlikely to reflect only reading failure and may play a causal role in developmental dyslexia.

Coordinates of hypoactivity in readers with dyslexia from the meta-analysis by Richlan et al. [83] can be quite closely matched with published coordinates of not just character-selective regions but also with other functionally defined regions of the ventral visual stream, such as general object-selective regions [93], limb- or body-selective regions [94], and face-selective regions [95]. The VWFA is surrounded by these high-level visual regions (see Figure 2), the closest neighbors being the limb-selective fusiform body area (FBA/OTS-limbs), the object-selective posterior fusiform/occipito-temporal sulcus (pFus/OTS), the ventral part of the lateral occipital complex or LOC) with which the VWFA partially overlaps, and the posterior fusiform face-selective region (pFus-faces/FFA-1) [37] of the left hemisphere. Functional abnormalities in the ventral visual stream of readers with dyslexia might therefore not be restricted to character-selective regions.

The meta-analysis of Richlan et al. [83] on functional brain abnormalities in people with dyslexia nonetheless only included studies if reading or reading-related tasks were performed and stimuli were letters or letter strings in an alphabetic script, which could be either words or pseudowords. When other objects have been used, results have sometimes been attributed to difficulties that are not strictly visual. For example, reduced activation in a left occipito-temporal area of dyslexic readers compared to typical readers is seen in picture naming relative to saying “yes” or “okay yes” to nonsense shapes, which was interpreted as a problem with visual-phonological integration [96]. Reduced BOLD activation in the bilateral ventral visual stream has also been reported in dyslexic adults while viewing an unfamiliar speaking or moving face [97]. The authors speculate that this could indicate a deficit in extracting face information that is needed to integrate visual and auditory information in natural speech perception.

However, the underactivation of the visual word form area has also been seen with visual stimuli that are hard (but not impossible) to verbalize, such as by the use of symbols in a visual search task [98]. A bilateral reduced fMRI adaptation has also been found for repeated objects and faces under passive viewing conditions in dyslexic compared to typical readers, including non-existent adaptation for faces in the fusiform face area (FFA) for the former group [99]. As all faces were unfamiliar—and therefore presumably un-nameable—it is hard to attribute these results to subvocalization or other verbal processes. It is also of note that Monzalvo et al. [60] found that children with dyslexia showed reduced activity for words in the left VWFA and for faces in the right fusiform face area (FFA) and a medial left ventral stream region (no particular group differences were found for responses to checkerboards or houses). In alignment with the neuronal recycling hypothesis, Monzalvo et al. [60] interpret this as a literacy-driven effect, where in the process of learning how to read, the area in the left hemisphere that becomes the VWFA in typical readers is recycled for the purpose of recognizing written words. Representations of visual words are suggested to compete with the representation of faces in the left fusiform gyrus, partially displacing face responses toward similar right hemisphere regions (for laterality effects in face perception, see [100]). However, while the FFA of typical readers (presumably with considerable reading experience) responds more to faces than the same region in readers with dyslexia (presumably with less reading experience), the left FFA in readers with dyslexia does not respond more to faces than the corresponding region in typical readers, even though that would be expected if left hemisphere face processing had less competition from word processing; in fact, the face responses of the left FFA of dyslexic readers are numerically lower than in typical readers [60]. An alternative interpretation of these data is that high-level visual processing of not just words but also other objects such as faces is unusual or deficient in developmental dyslexia. Important evidence comes from the aforementioned study by Centanni et al. [92] on at-risk children who later turned out to have dyslexia. These children not only showed left fusiform hypoactivity in response to letters, but also to pseudo-fonts and faces. Importantly, these functional abnormalities were found while the children were still in kindergarten. This may not only indicate an impairment of high-level visual mechanisms in dyslexia that are not specific to print, but that these impairments could be causal and not just an effect of lifelong problems with reading.

In sum, functional abnormalities of high-level regions within the ventral visual stream of readers with dyslexia are found across languages, could precede apparent reading problems, could reflect deficient visual processing, might not be restricted to character-selective regions, and may extend to objects other than words.

### 5.2. Structural

Functional differences in high-level regions of the ventral visual stream are consistently found between people with and without dyslexia, but the evidence for structural differences in these same regions is more mixed. Both reduced [101,102] and increased [103] cortical thickness in or around high-level ventral visual regions that selectively respond to written words have been reported; the discrepancies between the studies are not clear. Gray matter differences within bilateral fusiform gyri have also been used to classify people as dyslexic or typical readers using machine learning techniques; these authors report increased gray matter volume in these regions in readers with dyslexia [104]. A meta-analysis of voxel-based morphometry (VBM) studies nonetheless found that the largest cluster of grey matter reduction in relation to dyslexia was in the left occipito-temporal cortex consisting of mainly the fusiform gyrus and extending laterally into the inferior temporal gyrus [105]. Such structural abnormalities were furthermore found to overlap with functional underactivation in the left fusiform gyrus [105]. Furthermore, genetic carriers of the deletion 15q11.2 (BP1–BP2) both show an increased risk for developmental dyslexia as well as a smaller and less word-selective left fusiform gyrus [106].

However, a large-scale VBM study did not find group differences in gray matter volume [107]. Another meta-analysis also failed to identify consistent gray matter abnormalities in left occipito-temporal regions of people with dyslexia [108]. The authors point out that four out of the nine studies included in the meta-analysis did indeed find reduced gray matter volume in left ventral occipito-temporal regions, both the inferior temporal and fusiform gyri, but the peaks from these four studies might have been too scattered to be consistently revealed in the meta-analysis [108]. Even if structural changes accompany dyslexia, these might not necessarily play any causal role and could instead be a direct result of the different reading experience of dyslexic and non-dyslexic readers. For example, brain volume can change due to experience, including experience with reading [109,110,111]. Very interestingly, grey-matter reductions are found in left occipito-temporal/fusiform gyrus regions of preliterate children at familial risk for dyslexia [112,113]. It is therefore at least plausible that structural abnormalities of high-level regions in the ventral visual stream can causally contribute to reading problems in dyslexia.

### 5.3. EEG and MEG

**Figure 3:**
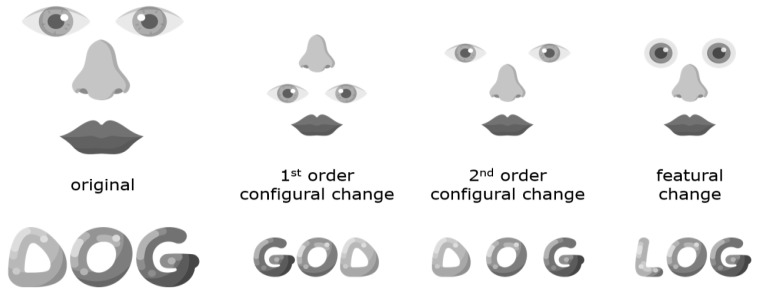
Maurer et al. [119] distinguish between three types of configural processing: sensitivity to first-order relations (relative position of features), holistic processing (gluing features into a gestalt or whole), and sensitivity to second-order relations (absolute distances between features). Featural processing has been used to describe processing individual object components or parts. Different manipulations of visual stimuli have been used to encourage the use of these types of visual processes (for a critical discussion on visual processes and how to measure them, see e.g., [120,121]. Recognition by parts as well as whole word shape have been suggested to independently contribute to reading, although the former likely plays a greater role than the latter [122]. Icons by Flaticon.com, (accessed on 20 September 2021).

For obvious reasons, electroencephalography (EEG) research on dyslexia has focused on the processing of text, and the literature is far too large to review here. However, we will review some of the literature on the N170 for words, as well as some work on visual objects such as faces. The N170 is a visual event-related potential (ERP) component of negative polarity that peaks around 170 ms after stimulus onset. A negative ERP peaking at this time point can be triggered by several different visual stimuli, but the N170 amplitude tends to be particularly large for words and faces. It has been suggested that the N170 component, at least when it comes to faces, reflects perceptual pre-categorical structural encoding as opposed to subsequent processes that utilize this structural description for recognition and identification [114]. While it has furthermore been proposed [114] to reflect configural processing (e.g., holistic or global configurations of whole faces) but not the processing of features (e.g., eyes), the N170 component likely taps into both configural and feature-based processing [115,116,117] (see Figure 3). N170 for words has also been suggested to reflect prelexical visual processes, which could be relatively more global or holistic for frequently encountered words [118].

Typical readers show a larger N170 to words or word-like stimuli than to other visual stimuli (e.g., symbol strings) when measured over left occipito-temporal regions, as well as a larger N170 for faces compared to several other object categories, often bilaterally but especially over right occipito-temporal sites [123,124,125,126,127,128,129]. The left lateralization of neural responses to print can even be found in children after only 2–3 months of formal reading instruction [130], but see [131] for contradictory evidence. The N170 has significant sources in high-level regions of the ventral stream. The N170 response to faces as well as its corresponding magnetoencephalographic (MEG) component M170 co-localize in the middle to posterior fusiform gyrus [132] although sources can be task-dependent [133]. Early N170 word tuning (words > symbols) has significant sources in the left temporal-parietal-occipital junction [125]. These sources appear to partly overlap with the object-selective lateral occipital complex [134]. Late N170 tuning has sources near the left fusiform gyrus [125].

Readers with dyslexia have an abnormal N170 tuning for print. Larger left occipito-temporal N170 potentials were found in response to word-like stimuli than to symbol strings in adult typical readers, but no such N170 tuning for print was found in adults with severe reading deficits [135]. An earlier MEG study found comparable results [136]. N170 word tuning is also found in typically reading but not children with dyslexia, although the developmental trajectory of N170 word tuning might be non-linear [126,137,138,139]. While this has been interpreted as a link to a core phonological deficit in dyslexia, importantly, N170 print tuning (words vs. false-font strings) in beginning readers is related to reading speed and vocabulary but unrelated to measures of phonological processing [140]. This is an interesting fact as a phonological processing deficit is often considered the primary causal factor of developmental dyslexia [1,4,5,141,142,143,144]. A parsimonious account of faulty print tuning in dyslexia is that it reflects faulty visual processing of print. This could be an effect of problematic reading and not its cause, as visual experience with individuating objects of particular categories clearly shapes the workings of the visual system [145]. Even adult non-dyslexic poor readers, however, show N170 tuning for word-like stimuli compared to symbol strings while readers with dyslexia matched for reading level lack such tuning [146]. Impaired N170 print tuning might thus be specific to dyslexia and not just a correlate of low reading skills per se or a lack of reading experience.

While many EEG studies on reading problems have measured ERPs such as the N170, where EEG signals triggered by specific events are averaged over multiple trials, the EEG signal can also be rhythmically perturbed through fast periodic visual stimulation. van de Walle de Ghelcke et al. [147] used this method to measure selective neural responses to letter strings in first graders. Letter strings were inserted periodically in pseudo-fonts, where one in five strings in a sequence was composed of real letters. The letter strings were real words that the children had been taught to recognize globally, real words learned through the phonics method that emphasizes grapheme-phoneme mapping, and pseudowords that also are thought to tap into grapheme-phoneme mapping. Responses were left-lateralized for pseudowords and words learned through phonics, but bilateral for words learned globally, indicating that neural responses are influenced by how the mapping of the written word is formed. This is also consistent with the well-established right hemisphere laterality of holistic or global processing and the left hemisphere laterality for feature-based processing [148] (see Figure 3). This bilateral response to globally learned words was most prominent for poor readers, consistent with an increased tendency to process words globally or holistically rather than by features.

Unsurprisingly, fewer studies on dyslexic readers have focused on evoked responses to objects than to print. Mayseless and Breznitz [149] asked dyslexic and typical readers to perform an object decision task, where participants indicated whether images depicted real or fake objects. Readers with dyslexia showed shorter ERP latencies than typical readers as well as a different brain activation pattern that appeared at an early processing state, suggesting that people with dyslexia process visual objects differently than typical readers. The authors speculated that shorter ERP latencies could reflect a holistic processing strategy in readers with dyslexia, which fits with possible weaknesses in feature-based visual processing, as further discussed in the chapter on behavioral evidence below. A diminished N170 in dyslexic readers has also been demonstrated for a moving or speaking face [150]. However, as participants had to report what word they had understood, the unusual neural processing for faces might reflect deficient verbal processing.

In contrast, readers with dyslexia who performed a face recognition task showed a normal N170 for faces, leading those authors to conclude that face perception is intact in dyslexia; the authors furthermore suggested that the deficits of recognition memory previously reported in readers with dyslexia is probably specific to verbal material [151]. Tarkiainen et al. [152], on the other hand, did find evidence for impaired face recognition in dyslexic readers (see also behavioral evidence subchapter below). Despite this, Tarkiainen et al. [152] found no apparent deficits in neural processing for faces in readers with dyslexia. Tarkiainen et al. [152] could not detect significant differences in the MEG activation of occipital and occipito-temporal regions between the groups when faces and other objects were shown. They concluded that early visual analysis and processing of features and faces are essentially normal in people with dyslexia, and that the occipito-temporal dysfunction in dyslexic individuals is largely specific to letter-string processing.

The results of Rüsseler et al. [151] and Tarkiainen et al. [152] could be true null effects. There are, however, other possibilities. Both studies had relatively small samples (12 readers with dyslexia in Rüsseler et al. [151], 8 readers with dyslexia in Tarkiainen et al. [152]) so any group differences would be hard to detect. The null results of Tarkiainen et al. [152] might also be due to the fact that in the MEG part of the study, the task of the subjects was not to identify faces but to name the facial expression (e.g., “happy”) when prompted. A large body of previous research has shown that judging the identity of a face and judging its expression rely on neural processes that are largely separable [153,154]. The task used by Tarkiainen et al. [152] might not have sufficiently tapped into the neural processes that support individuation. Finally, Rüsseler et al. [151] used photographs of natural faces (as opposed to e.g., sketches) which may have triggered a holistic visual process which could inhibit feature-based processing [116]. As holistic processing of faces might be intact in developmental dyslexia while feature-based processing could be impaired (see behavioral evidence subchapter), it is possible that N170 differences in face processing are primarily detectable under circumstances where feature-based processing of faces is necessary.

While the overall amplitude of N170 for faces could be similar in dyslexic and typical readers, the component’s laterality might differ between the two groups. Typical readers showed an expected left lateralization for the N170 triggered by words and right lateralization for the N170 for faces, but people with developmental dyslexia showed no signs of laterality for either category [155]. This is in accordance with the possibility that literacy leads to competition for neural resources between words and faces in high-level ventral stream regions of the left hemisphere. It should be emphasized that literacy-driven competition between faces and words and faulty high-level visual processing in readers with dyslexia are not mutually exclusive possibilities. Indeed, Collins et al. [155] reported face processing deficits in their participants with dyslexia as measured behaviorally. We now turn to such behavioral work on potential high-level visual problems in developmental dyslexia.

## 6. Behavioral Evidence

There is some behavioral evidence arguing for visual object processing difficulties of readers with dyslexia. The evidence mostly comes from behavioral studies on faces, but a few studies have also been conducted on other objects.

It is well-established that readers with dyslexia are slower than readers without dyslexia in naming pictures of objects (e.g., [15,156]). This has been attributed to a problem in automatization of verbal responses to visual stimuli [15]. Readers with dyslexia were also found to be more error-prone at naming objects compared to matched participants with other problems (e.g., ADHD, poor handwriting, dyscalculia) with an effect size that was large (Cohen’s d = 0.936) and close to significance (*p* = 0.05); reading age but not chronological age furthermore correlated with naming accuracy [157]. However, the errors that the readers with dyslexia made indicated that they might have recognized the objects but just did not find the proper names for them (e.g., saying staxaphone or styraphone when shown a xylophone), leading the authors to suggest that object naming problems are not indicative of a perceptual impairment.

However, tasks that measure rapid automatized naming of objects often require only basic-level categorization (e.g., octopus, microscope [157]) while word recognition requires subordinate-level categorization. To rephrase, it is not enough to recognize that something is a word, one has to individuate words, many of which are very similar (e.g., mat, map, mad, dam, bam). Basic-level object recognition might not suffice to reveal subtle high-level visual problems in readers with dyslexia. Individuating objects, such as being able to tell two different octopuses or two similar-looking microscopes apart, should put more demands on fine-grained analysis of shape that likely supports visual word identification.

Indeed, Sigurdardottir et al. [95] found that readers with dyslexia did significantly worse on the Vanderbilt Expertise Test [158] than matched controls, a task that required the individuation of same-category non-face objects (different types of birds, butterflies, cars, houses, and planes), but found no differences on a color recognition task that did not require any shape analysis. Huestegge et al. [159] also found that readers with dyslexia remembered complex abstract figures in less detail (akin to subordinate-level recognition) but were no different from or even slightly better than controls at recognizing them on a basic level. The authors attribute this to greater processing of whole objects at the expense of diminished processing of visual details. Interestingly, detail-related errors in the dyslexic group were completely uncorrelated with measures of phonological skills which could indicate that phonological processing and high-level visual processing could independently contribute to reading problems. Readers with dyslexia have also been found to be slower than controls at deciding whether visual stimuli are real or fake objects [149] and are less accurate at telling apart real and fake traffic signs [160]. However, Gabay et al. [161] found no differences between the ability of dyslexic and typical readers to tell different cars apart, and neither did Sigurdardottir et al. [162] for the ability to tell similar-looking computer-generated novel objects apart.

Results on face processing abilities of readers with dyslexia have also been mixed. Some studies find no particular problems [151,163,164,165]. Such null results could be due to the usefulness of low-level visual characteristics or other cues such as hairstyle or hair length that are unrelated to face identification in the chosen tasks. They can also reflect the heterogeneity of developmental dyslexia. For example, Kühn et al. [166] studied 24 high school students with developmental dyslexia. Face recognition deficits were not found on the group level, but some individuals with dyslexia nonetheless had difficulties with face recognition, while there was a dissociation between reading abilities and face recognition of other readers with dyslexia. Face recognition deficits can therefore be present in dyslexia, but they are not universal for the dyslexic population, arguing for individual differences in dyslexia.

Several studies have reported abnormal face processing abilities of readers with dyslexia (see e.g., [92,95,152,155,161,162,167,168,169,170,171]). For example, Tarkiainen et al. [152] showed that readers with dyslexia made more errors than control participants on the Benton facial recognition test [167] where participants had to match either identical images or two images from different viewpoints of the same person. Readers with dyslexia were also slower in a computerized matching task where participants were asked to match one of two faces in the lower half of the computer screen to a reference face shown in the upper half of the screen. Sigurdardottir et al. [95] reported that readers with dyslexia performed significantly worse than matched controls on the Cambridge Face Memory Test (CFMT) that requires the recognition of individual faces [172]. Furthermore, face matching performance was found to predict dyslexia over and above the matching of novel objects or of noise pattern that shared low-level visual characteristics with the faces such as orientation or spatial frequency information [162]. This supports that the association between word and face processing is quite specific. Face processing problems of readers with dyslexia are also not associated with dyslexic readers’ verbal deficits, as assessed by verbal working memory [168]. High-level visual problems in readers with dyslexia might therefore be independent of a phonological processing deficit commonly seen as the primary cause of dyslexia. Lastly, Centanni et al. [92] studied children at risk of developing dyslexia. They found performance differences in a one-back face recognition task, distinguishing between at-risk children who later turned out to have dyslexia and those at-risk children who did not. Finding these behavioral differences in children before the beginning of formal reading instruction suggests that high-level visual processing deficits may be causal to dyslexia as opposed to an effect of a diminished exposure to written words.

We initially expected these problems in dyslexia to reflect a problem with visual learning [50], leading to recognition problems that were particularly great for highly familiar object categories such as faces and words that depend on such learning [145,162], but did not find support for such a visual expertise account of dyslexia [168]. This unforeseen result pointed us in a different direction, namely, to focus on the type of visual characteristics with which readers with dyslexia might struggle and the neural mechanisms that support their processing. Holistic and featural processing may provide two different pathways to recognition [173,174] (see Figure 3). Although holistic processing of words contributes somewhat to reading, feature-based processing of smaller word parts appears to be much more important [122]. Our research suggests that dyslexic readers show problems with matching faces based on their features but not their global form [121], and their configural or holistic processing of faces seems to be intact [95]. Follow-up work suggests that dyslexic readers depend on only a single visual process regardless of whether features or configurations are task-relevant [175]. We speculate that this single visual process is holistic rather than featural and suggest that behavioral manifestations of high-level visual problems in developmental dyslexia become apparent when featural processing is particularly beneficial for object individuation and recognition, such as in visual word recognition.

## 7. Practical Implications

In practice, it is possible that detecting high-level visual impairments could be used for early diagnosis of dyslexia, as well as improving reading abilities. For example, simple and quick visual perception tasks relying on high-level vision might be able to identify those at risk for developing a reading disability. This could include passive rapid viewing of objects at the subordinate or basic level, or of faces with different spatial frequency properties, combined with EEG measurements like SSVEP—Steady-State Visually Evoked Potential [176] (see our preregistration: https://osf.io/4dr3f/ accessed on 7 June 2021). In the cases where reading impairments can be attributed to a high-level visual deficit, people could be trained to adopt a different strategy for reading. It is, however, an empirical question, yet to be answered, of whether such interventions should focus on training readers with dyslexia to better use their impaired high-level visual processing, or on the contrary, focus on making further use of other unimpaired abilities. This would need to be studied and evaluated both for children and adults. As pointed out by Lochy et al. [177], early interventions in dyslexia result in better outcomes, so developing sensitive measures that might later even be applied before reading difficulties start to pose significant problems is of considerable practical value. We want to explicitly say, however, that while novel screening methods might be able to identify those at risk for developing a reading disability, they should never be put into common practice without strong empirical support. Similarly, while the high-level visual dysfunction hypothesis could in the future lead to novel training programs for children and adults who struggle with reading, these should never be applied instead of existing evidence-based methods without extensive further study, as this might end up doing more harm than good.

## 8. Conclusions

In the past few years, there has been increased interest in the potential role of visual processes in the ventral visual stream as a cause of reading deficits. The ventral view, which we also refer to as the high-level visual dysfunction hypothesis, predicts that reading deficits can stem from problems with specific visual object perception mechanisms. The ventral view is newly formed, understudied, and still so unknown within the dyslexia research community that it has not even had the chance to be (rightfully) skeptically received yet. Further empirical testing is greatly needed. Given the evidence provided, it should, however, at least be considered plausible that reading problems in dyslexia can in some cases be traced to the functioning of high-level visual mechanisms.

## Data Availability

Not applicable.

## References

[1] Catts H.W. (1989). Defining dyslexia as a developmental language disorder. Ann. Dyslexia.

[2] Pennington B.F., Van Orden G.C., Smith S.D., Green P.A., Haith M.M. (1990). Phonological processing skills and deficits in adult dyslexics. Child Dev..

[3] Shaywitz S.E., Shaywitz B.A. (2005). Dyslexia (specific reading disability). Biol. Psychiatry.

[4] Snowling M.J. (2001). From language to reading and dyslexia 1. Dyslexia.

[5] Vellutino F.R., Fletcher J.M., Snowling M.J., Scanlon D.M. (2004). Specific reading disability (dyslexia): What have we learned in the past four decades?. J. Child Psychol. Psychiatry.

[6] Lyon G.R., Shaywitz S.E., Shaywitz B.A. (2003). A definition of dyslexia. Ann. Dyslexia.

[7] Shaywitz B.A., Shaywitz S.E. (2020). The American experience: Towards a 21st century definition of dyslexia. Oxf. Rev. Educ..

[8] De Martino S., Espesser R., Rey V., Habib M. (2001). The “temporal processing deficit” hypothesis in dyslexia: New experimental evidence. Brain Cogn..

[9] Farmer M.E., Klein R.M. (1995). The evidence for a temporal processing deficit linked to dyslexia: A review. Psychon. Bull. Rev..

[10] Goswami U. (2011). A temporal sampling framework for developmental dyslexia. Trends Cogn. Sci..

[11] Giofrè D., Toffalini E., Provazza S., Calcagnì A., Altoè G., Roberts D.J. (2019). Are children with developmental dyslexia all the same? A cluster analysis with more than 300 cases. Dyslexia.

[12] Stein J. (2019). The current status of the magnocellular theory of developmental dyslexia. Neuropsychologia.

[13] Valdois S., Bosse M.L., Tainturier M.J. (2004). The cognitive deficits responsible for developmental dyslexia: Review of evidence for a selective visual attentional disorder. Dyslexia.

[14] Norton E.S., Beach S.D., Gabrieli J.D. (2015). Neurobiology of dyslexia. Curr. Opin. Neurobiol..

[15] Denckla M.B., Rudel R.G. (1976). Rapid ‘automatized’naming (RAN): Dyslexia differentiated from other learning disabilities. Neuropsychologia.

[16] Reid A.A. (2018). Neuroimaging reveals heterogeneous neural correlates of reading deficit in individuals with dyslexia consistent with a multiple deficit model. Neuroimaging-Structure, Function and Mind.

[17] O’Brien G., Yeatman J.D. (2021). Bridging sensory and language theories of dyslexia: Toward a multifactorial model. Dev. Sci..

[18] Ziegler J.C., Bertrand D., Tóth D., Csépe V., Reis A., Faísca L., Saine N., Lyytinen H., Vaessen A., Blomert L. (2010). Orthographic depth and its impact on universal predictors of reading: A cross-language investigation. Psychol. Sci..

[19] Cox D.D. (2014). Do we understand high-level vision?. Curr. Opin. Neurobiol..

[20] Cox D.D., Dean T. (2014). Neural networks and neuroscience-inspired computer vision. Curr. Biol..

[21] Kussmaul A. (1877). Disturbance of speech. Cyclopedia of the Practice of Medicine.

[22] Hinshelwood J. (1895). Word-blindness and visual memory. Lancet.

[23] Hinshelwood J. (1900). Congenital word-blindness. Lancet.

[24] Morgan W.P. (1896). A case of congenital word blindness. Br. Med. J..

[25] Papert S.A. The Summer Vision Project. https://dspace.mit.edu/handle/1721.1/6125.

[26] Cichy R.M., Kaiser D. (2019). Deep neural networks as scientific models. Trends Cogn. Sci..

[27] Glasser M.F., Coalson T.S., Robinson E.C., Hacker C.D., Harwell J., Yacoub E., Ugurbil K., Andersson J., Beckmann C.F., Jenkinson M. (2016). A multi-modal parcellation of human cerebral cortex. Nature.

[28] Felleman D.J., Van Essen D.C. (1991). Distributed hierarchical processing in the primate cerebral cortex. Cereb. Cortex.

[29] Brooks D., Sigurdardottir H.M., Sheinberg D.L. (2014). The neurophysiology of attention and object recognition in visual scenes. Scene Vision.

[30] Foster K., Gaska J.P., Nagler M., Pollen D. (1985). Spatial and temporal frequency selectivity of neurones in visual cortical areas V1 and V2 of the macaque monkey. J. Physiol..

[31] Hubel D.H., Wiesel T.N. (1968). Receptive fields and functional architecture of monkey striate cortex. J. Physiol..

[32] Shapley R., Kaplan E., Soodak R. (1981). Spatial summation and contrast sensitivity of X and Y cells in the lateral geniculate nucleus of the macaque. Nature.

[33] Goodale M.A., Milner A.D. (1992). Separate visual pathways for perception and action. Trends Neurosci..

[34] Pitcher D., Ungerleider L.G. (2021). Evidence for a third visual pathway specialized for social perception. Trends Cogn. Sci..

[35] Ungerleider L.G., Haxby J.V. (1994). ‘What’and ‘where’ in the human brain. Curr. Opin. Neurobiol..

[36] Ungerleider L.G., Mishkin M., Goodale M., Ingle D.J., Mansfield R.J.W. (1982). Two cortical visual systems. Analysis of Visual Behavior.

[37] Grill-Spector K., Weiner K.S. (2014). The functional architecture of the ventral temporal cortex and its role in categorization. Nature Rev. Neurosci..

[38] Desimone R., Albright T.D., Gross C.G., Bruce C. (1984). Stimulus-selective properties of inferior temporal neurons in the macaque. J. Neurosci..

[39] Gross C.G., Rocha-Miranda C.d., Bender D. (1972). Visual properties of neurons in inferotemporal cortex of the macaque. J. Neurophysiol..

[40] Milner D., Goodale M. (2006). The Visual Brain in Action.

[41] Logothetis N.K., Sheinberg D.L. (1996). Visual object recognition. Annu. Rev. Neurosci..

[42] Palmeri T.J., Gauthier I. (2004). Visual object understanding. Nat. Rev. Neurosci..

[43] Tanaka K., Saito H.-a., Fukada Y., Moriya M. (1991). Coding visual images of objects in the inferotemporal cortex of the macaque monkey. J. Neurophysiol..

[44] Kar K., DiCarlo J.J. (2021). Fast recurrent processing via ventrolateral prefrontal cortex is needed by the primate ventral stream for robust core visual object recognition. Neuron.

[45] Kietzmann T.C., Spoerer C.J., Sörensen L.K., Cichy R.M., Hauk O., Kriegeskorte N. (2019). Recurrence is required to capture the representational dynamics of the human visual system. Proc. Natl. Acad. Sci. USA.

[46] Dehaene S., Cohen L., Sigman M., Vinckier F. (2005). The neural code for written words: A proposal. Trends Cogn. Sci..

[47] Anzai A., Peng X., Van Essen D.C. (2007). Neurons in monkey visual area V2 encode combinations of orientations. Nat. Neurosci..

[48] Pegado F., Nakamura K., Cohen L., Dehaene S. (2011). Breaking the symmetry: Mirror discrimination for single letters but not for pictures in the Visual Word Form Area. Neuroimage.

[49] Zhou Z., Vilis T., Strother L. (2019). Functionally separable font-invariant and font-sensitive neural populations in occipitotemporal cortex. J. Cogn. Neurosci..

[50] Sigurdardottir H.M., Danielsdottir H.B., Gudmundsdottir M., Hjartarson K.H., Thorarinsdottir E.A., Kristjánsson Á. (2017). Problems with visual statistical learning in developmental dyslexia. Sci. Rep..

[51] Groen I.I., Silson E.H., Baker C.I. (2017). Contributions of low-and high-level properties to neural processing of visual scenes in the human brain. Philos. Trans. R. Soc. B Biol. Sci..

[52] Vinckier F., Dehaene S., Jobert A., Dubus J.P., Sigman M., Cohen L. (2007). Hierarchical coding of letter strings in the ventral stream: Dissecting the inner organization of the visual word-form system. Neuron.

[53] Cohen L., Henry C., Dehaene S., Martinaud O., Lehéricy S., Lemer C., Ferrieux S. (2004). The pathophysiology of letter-by-letter reading. Neuropsychologia.

[54] Leff A., Spitsyna G., Plant G., Wise R. (2006). Structural anatomy of pure and hemianopic alexia. J. Neurol. Neurosurg. Psychiatry.

[55] Pflugshaupt T., Gutbrod K., Wurtz P., von Wartburg R., Nyffeler T., de Haan B., Karnath H.-O., Mueri R.M. (2009). About the role of visual field defects in pure alexia. Brain.

[56] Mani J., Diehl B., Piao Z., Schuele S., Lapresto E., Liu P., Nair D., Dinner D., Lüders H. (2008). Evidence for a basal temporal visual language center: Cortical stimulation producing pure alexia. Neurology.

[57] Dehaene S., Cohen L. (2011). The unique role of the visual word form area in reading. Trends Cogn. Sci..

[58] Dundas E.M., Plaut D.C., Behrmann M. (2013). The joint development of hemispheric lateralization for words and faces. J. Exp. Psychol. Gen..

[59] Dundas E.M., Plaut D.C., Behrmann M. (2014). An ERP investigation of the co-development of hemispheric lateralization of face and word recognition. Neuropsychologia.

[60] Monzalvo K., Fluss J., Billard C., Dehaene S., Dehaene-Lambertz G. (2012). Cortical networks for vision and language in dyslexic and normal children of variable socio-economic status. Neuroimage.

[61] Dehaene S., Cohen L., Morais J., Kolinsky R. (2015). Illiterate to literate: Behavioural and cerebral changes induced by reading acquisition. Nat. Rev. Neurosci..

[62] Caspers J., Zilles K., Eickhoff S.B., Schleicher A., Mohlberg H., Amunts K. (2013). Cytoarchitectonical analysis and probabilistic mapping of two extrastriate areas of the human posterior fusiform gyrus. Brain Struct. Funct..

[63] Cohen L., Dehaene S., Naccache L., Lehéricy S., Dehaene-Lambertz G., Hénaff M.-A., Michel F. (2000). The visual word form area: Spatial and temporal characterization of an initial stage of reading in normal subjects and posterior split-brain patients. Brain.

[64] Cohen L., Lehéricy S., Chochon F., Lemer C., Rivaud S., Dehaene S. (2002). Language-specific tuning of visual cortex? Functional properties of the Visual Word Form Area. Brain.

[65] Price C.J., Devlin J.T. (2003). The myth of the visual word form area. Neuroimage.

[66] Binder J.R., Medler D.A., Westbury C.F., Liebenthal E., Buchanan L. (2006). Tuning of the human left fusiform gyrus to sublexical orthographic structure. Neuroimage.

[67] Güçlü U., van Gerven M.A. (2015). Deep neural networks reveal a gradient in the complexity of neural representations across the ventral stream. J. Neurosci..

[68] Stigliani A., Weiner K.S., Grill-Spector K. (2015). Temporal processing capacity in high-level visual cortex is domain specific. J. Neurosci..

[69] Dien J. (2009). A tale of two recognition systems: Implications of the fusiform face area and the visual word form area for lateralized object recognition models. Neuropsychologia.

[70] Barton J.J., Fox C.J., Sekunova A., Iaria G. (2010). Encoding in the visual word form area: An fMRI adaptation study of words versus handwriting. J. Cogn. Neurosci..

[71] Bouhali F., de Schotten M.T., Pinel P., Poupon C., Mangin J.-F., Dehaene S., Cohen L. (2014). Anatomical connections of the visual word form area. J. Neurosci..

[72] Song Y., Bu Y., Hu S., Luo Y., Liu J. (2010). Short-term language experience shapes the plasticity of the visual word form area. Brain Res..

[73] Turk-Browne N.B., Scholl B.J., Chun M.M., Johnson M.K. (2009). Neural evidence of statistical learning: Efficient detection of visual regularities without awareness. J. Cogn. Neurosci..

[74] Reinke K., Fernandes M., Schwindt G., O’Craven K., Grady C.L. (2008). Functional specificity of the visual word form area: General activation for words and symbols but specific network activation for words. Brain Lang..

[75] Dehaene S., Pegado F., Braga L., Ventura Filho P., GN J. (2010). Impact of literacy on the cortical networks for vision and language. Science.

[76] Nestor A., Behrmann M., Plaut D.C. (2013). The neural basis of visual word form processing: A multivariate investigation. Cereb. Cortex.

[77] Starrfelt R., Gerlach C. (2007). The visual what for area: Words and pictures in the left fusiform gyrus. Neuroimage.

[78] Gauthier I., Tarr M.J., Moylan J., Anderson A.W., Skudlarski P., Gore J.C. (2000). Does visual subordinate-level categorisation engage the functionally defined fusiform face area?. Cogn. Neuropsychol..

[79] Kosslyn S.M., Alpert N.M., Thompson W.L. (1995). Identifying objects at different levels of hierarchy: A positron emission tomography study. Hum. Brain Mapp..

[80] Rogers T.T., Hocking J., Mechelli A., Patterson K., Price C. (2005). Fusiform activation to animals is driven by the process, not the stimulus. J. Cogn. Neurosci..

[81] Borghesani V., Wang C., Watson C., Bouhali F., Caverzasi E., Battistella G., Bogley R., Yabut N.A., Deleon J., Miller Z.A. (2021). Functional and morphological correlates of developmental dyslexia: A multimodal investigation of the ventral occipitotemporal cortex. J. Neuroimaging.

[82] Brem S., Maurer U., Kronbichler M., Schurz M., Richlan F., Blau V., Reithler J., van der Mark S., Schulz E., Bucher K. (2020). Visual word form processing deficits driven by severity of reading impairments in children with developmental dyslexia. Sci. Rep..

[83] Richlan F., Kronbichler M., Wimmer H. (2011). Meta-analyzing brain dysfunctions in dyslexic children and adults. Neuroimage.

[84] Kronbichler L., Kronbichler M. (2018). The importance of the left occipitotemporal cortex in developmental dyslexia. Curr. Dev. Disord. Rep..

[85] Banfi C., Koschutnig K., Moll K., Schulte-Körne G., Fink A., Landerl K. (2021). Reading-related functional activity in children with isolated spelling deficits and dyslexia. Lang. Cogn. Neurosci..

[86] Dębska A., Banfi C., Chyl K., Dzięgiel-Fivet G., Kacprzak A., Łuniewska M., Plewko J., Grabowska A., Landerl K., Jednoróg K. (2021). Neural patterns of word processing differ in children with dyslexia and isolated spelling deficit. Brain Struct. Funct..

[87] Cutting L.E., Clements-Stephens A., Pugh K.R., Burns S., Cao A., Pekar J.J., Davis N., Rimrodt S.L. (2013). Not all reading disabilities are dyslexia: Distinct neurobiology of specific comprehension deficits. Brain Connect..

[88] Martin A., Kronbichler M., Richlan F. (2016). Dyslexic brain activation abnormalities in deep and shallow orthographies: A meta-analysis of 28 functional neuroimaging studies. Hum. Brain Mapp..

[89] Liu L., Wang W., You W., Li Y., Awati N., Zhao X., Booth J.R., Peng D. (2012). Similar alterations in brain function for phonological and semantic processing to visual characters in Chinese dyslexia. Neuropsychologia.

[90] Hu W., Lee H.L., Zhang Q., Liu T., Geng L.B., Seghier M.L., Shakeshaft C., Twomey T., Green D.W., Yang Y.M. (2010). Developmental dyslexia in Chinese and English populations: Dissociating the effect of dyslexia from language differences. Brain.

[91] Raschle N.M., Zuk J., Gaab N. (2012). Functional characteristics of developmental dyslexia in left-hemispheric posterior brain regions predate reading onset. Proc. Natl. Acad. Sci. USA.

[92] Centanni T.M., Norton E.S., Ozernov-Palchik O., Park A., Beach S.D., Halverson K., Gaab N., Gabrieli J.D. (2019). Disrupted left fusiform response to print in beginning kindergartners is associated with subsequent reading. NeuroImage Clin..

[93] Grill-Spector K., Kushnir T., Hendler T., Malach R. (2000). The dynamics of object-selective activation correlate with recognition performance in humans. Nat. Neurosci..

[94] Vocks S., Busch M., Grönemeyer D., Schulte D., Herpertz S., Suchan B. (2010). Differential neuronal responses to the self and others in the extrastriate body area and the fusiform body area. Cogn. Affect. Behav. Neurosci..

[95] Sigurdardottir H.M., Ívarsson E., Kristinsdóttir K., Kristjánsson Á. (2015). Impaired recognition of faces and objects in dyslexia: Evidence for ventral stream dysfunction?. Neuropsychology.

[96] McCrory E.J., Mechelli A., Frith U., Price C.J. (2005). More than words: A common neural basis for reading and naming deficits in developmental dyslexia?. Brain.

[97] Rüsseler J., Ye Z., Gerth I., Szycik G.R., Münte T.F. (2018). Audio-visual speech perception in adult readers with dyslexia: An fMRI study. Brain Imaging Behav..

[98] Boros M., Anton J.-L., Pech-Georgel C., Grainger J., Szwed M., Ziegler J.C. (2016). Orthographic processing deficits in developmental dyslexia: Beyond the ventral visual stream. NeuroImage.

[99] Perrachione T.K., Del Tufo S.N., Winter R., Murtagh J., Cyr A., Chang P., Halverson K., Ghosh S.S., Christodoulou J.A., Gabrieli J.D. (2016). Dysfunction of rapid neural adaptation in dyslexia. Neuron.

[100] Sigurdardottir H.M., Jozranjbar B., Vonk J., Shackelford T. (2019). Laterality effect (face perception). Encyclopedia of Animal Cognition and Behavior.

[101] Altarelli I., Monzalvo K., Iannuzzi S., Fluss J., Billard C., Ramus F., Dehaene-Lambertz G. (2013). A functionally guided approach to the morphometry of occipitotemporal regions in developmental dyslexia: Evidence for differential effects in boys and girls. J. Neurosci..

[102] Adrián-Ventura J., Soriano-Ferrer M., Fuentes-Claramonte P., Morte-Soriano M., Parcet M.A., Avila C. (2020). Grey matter reduction in the occipitotemporal cortex in Spanish children with dyslexia: A voxel-based morphometry study. J. Neurolinguistics.

[103] Ma Y., Koyama M.S., Milham M.P., Castellanos F.X., Quinn B.T., Pardoe H., Wang X., Kuzniecky R., Devinsky O., Thesen T. (2015). Cortical thickness abnormalities associated with dyslexia, independent of remediation status. NeuroImage Clin..

[104] Tamboer P., Vorst H., Ghebreab S., Scholte H. (2016). Machine learning and dyslexia: Classification of individual structural neuro-imaging scans of students with and without dyslexia. NeuroImage Clin..

[105] Linkersdörfer J., Lonnemann J., Lindberg S., Hasselhorn M., Fiebach C.J. (2012). Grey matter alterations co-localize with functional abnormalities in developmental dyslexia: An ALE meta-analysis. PLoS ONE.

[106] Ulfarsson M., Walters G., Gustafsson O., Steinberg S., Silva A., Doyle O., Brammer M., Gudbjartsson D., Arnarsdottir S., Jonsdottir G. (2017). 15q11. 2 CNV affects cognitive, structural and functional correlates of dyslexia and dyscalculia. Transl. Psychiatry.

[107] Jednorog K., Marchewka A., Altarelli I., Monzalvo Lopez A.K., van Ermingen-Marbach M., Grande M., Grabowska A., Heim S., Ramus F. (2015). How reliable are gray matter disruptions in specific reading disability across multiple countries and languages? Insights from a large-scale voxel-based morphometry study. Hum. Brain Mapp..

[108] Richlan F., Kronbichler M., Wimmer H. (2013). Structural abnormalities in the dyslexic brain: A meta-analysis of voxel-based morphometry studies. Hum. Brain Mapp..

[109] Carreiras M., Seghier M.L., Baquero S., Estévez A., Lozano A., Devlin J.T., Price C.J. (2009). An anatomical signature for literacy. Nature.

[110] Krafnick A.J., Flowers D.L., Luetje M.M., Napoliello E.M., Eden G.F. (2014). An investigation into the origin of anatomical differences in dyslexia. J. Neurosci..

[111] Beelen C., Blockmans L., Wouters J., Ghesquière P., Vandermosten M. (2021). Brain-behavior dynamics between the left fusiform and reading. Brain Struct. Funct..

[112] Raschle N.M., Chang M., Gaab N. (2011). Structural brain alterations associated with dyslexia predate reading onset. Neuroimage.

[113] Beelen C., Vanderauwera J., Wouters J., Vandermosten M., Ghesquière P. (2019). Atypical gray matter in children with dyslexia before the onset of reading instruction. Cortex.

[114] Eimer M. (2000). The face-specific N170 component reflects late stages in the structural encoding of faces. Neuroreport.

[115] Eimer M., Gosling A., Nicholas S., Kiss M. (2011). The N170 component and its links to configural face processing: A rapid neural adaptation study. Brain Res..

[116] Sagiv N., Bentin S. (2001). Structural encoding of human and schematic faces: Holistic and part-based processes. J. Cogn. Neurosci..

[117] Harris A., Nakayama K. (2008). Rapid adaptation of the M170 response: Importance of face parts. Cereb. Cortex.

[118] Simon G., Petit L., Bernard C., Rebaï M. (2007). N170 ERPs could represent a logographic processing strategy in visual word recognition. Behav. Brain Funct..

[119] Maurer D., Le Grand R., Mondloch C.J. (2002). The many faces of configural processing. Trends Cogn. Sci..

[120] Richler J., Palmeri T.J., Gauthier I. (2012). Meanings, mechanisms, and measures of holistic processing. Front. Psychol..

[121] Sigurdardottir H.M., Arnardottir A., Halldorsdottir E.T., Omarsdottir H.R., Valgeirsdottir A.S. (2019). Faces and words are both associated and dissociated: Evidence from visual problems in dyslexia. PsyArXiv.

[122] Pelli D.G., Tillman K.A. (2007). Parts, wholes, and context in reading: A triple dissociation. PLoS ONE.

[123] Bentin S., Allison T., Puce A., Perez E., McCarthy G. (1996). Electrophysiological studies of face perception in humans. J. Cogn. Neurosci..

[124] Bentin S., Mouchetant-Rostaing Y., Giard M.-H., Echallier J.-F., Pernier J. (1999). ERP manifestations of processing printed words at different psycholinguistic levels: Time course and scalp distribution. J. Cogn. Neurosci..

[125] Brem S., Bucher K., Halder P., Summers P., Dietrich T., Martin E., Brandeis D. (2006). Evidence for developmental changes in the visual word processing network beyond adolescence. Neuroimage.

[126] Maurer U., McCandliss B.D. (2007). The development of visual expertise for words: The contribution of electrophysiology. Single-Word Reading.

[127] Rossion B. (2014). Understanding face perception by means of human electrophysiology. Trends Cogn. Sci..

[128] Rossion B., Jacques C. (2008). Does physical interstimulus variance account for early electrophysiological face sensitive responses in the human brain? Ten lessons on the N170. Neuroimage.

[129] Rossion B., Joyce C.A., Cottrell G.W., Tarr M.J. (2003). Early lateralization and orientation tuning for face, word, and object processing in the visual cortex. Neuroimage.

[130] van de Walle de Ghelcke A., Rossion B., Schiltz C., Lochy A. (2021). Developmental changes in neural letter-selectivity: A 1-year follow-up of beginning readers. Dev. Sci..

[131] Kast M., Elmer S., Jancke L., Meyer M. (2010). ERP differences of pre-lexical processing between dyslexic and non-dyslexic children. Int. J. Psychophysiol..

[132] Deffke I., Sander T., Heidenreich J., Sommer W., Curio G., Trahms L., Lueschow A. (2007). MEG/EEG sources of the 170-ms response to faces are co-localized in the fusiform gyrus. Neuroimage.

[133] Itier R.J., Taylor M.J. (2004). Source analysis of the N170 to faces and objects. Neuroreport.

[134] James T.W., Culham J., Humphrey G.K., Milner A.D., Goodale M.A. (2003). Ventral occipital lesions impair object recognition but not object-directed grasping: An fMRI study. Brain.

[135] Mahé G., Bonnefond A., Gavens N., Dufour A., Doignon-Camus N. (2012). Impaired visual expertise for print in French adults with dyslexia as shown by N170 tuning. Neuropsychologia.

[136] Helenius P., Tarkiainen A., Cornelissen P., Hansen P.C., Salmelin R. (1999). Dissociation of normal feature analysis and deficient processing of letter-strings in dyslexic adults. Cereb. Cortex.

[137] Maurer U., Schulz E., Brem S., van der Mark S., Bucher K., Martin E., Brandeis D. (2011). The development of print tuning in children with dyslexia: Evidence from longitudinal ERP data supported by fMRI. Neuroimage.

[138] Maurer U., Brem S., Kranz F., Bucher K., Benz R., Halder P., Steinhausen H.-C., Brandeis D. (2006). Coarse neural tuning for print peaks when children learn to read. Neuroimage.

[139] Fraga-González G., Pleisch G., Di Pietro S.V., Neuenschwander J., Walitza S., Brandeis D., Karipidis I.I., Brem S. (2021). The rise and fall of rapid occipito-temporal sensitivity to letters: Transient specialization through elementary school. Dev. Cogn. Neurosci..

[140] Eberhard-Moscicka A.K., Jost L.B., Raith M., Maurer U. (2015). Neurocognitive mechanisms of learning to read: Print tuning in beginning readers related to word-reading fluency and semantics but not phonology. Dev. Sci..

[141] Liberman I.Y., Shankweiler D., Liberman A.M., Shankweiler D., Liberman I.Y. (1989). The alphabetic principle and learning to read. Phonology and Reading Disability: Solving the Reading Puzzle.

[142] Peterson R.L., Pennington B.F. (2015). Developmental dyslexia. Annu. Rev. Clin. Psychol..

[143] Wagner R.K., Torgesen J.K. (1987). The nature of phonological processing and its causal role in the acquisition of reading skills. Psychol. Bull..

[144] Zoubrinetzky R., Bielle F., Valdois S. (2014). New insights on developmental dyslexia subtypes: Heterogeneity of mixed reading profiles. PLoS ONE.

[145] Sigurdardottir H.M., Gauthier I. (2015). Expertise and object recognition. Brain Mapp. Encycl. Ref..

[146] Mahé G., Bonnefond A., Doignon-Camus N. (2013). Is the impaired N170 print tuning specific to developmental dyslexia? A matched reading-level study with poor readers and dyslexics. Brain Lang..

[147] van de Walle de Ghelcke A., Rossion B., Schiltz C., Lochy A. (2020). Impact of learning to read in a mixed approach on neural tuning to words in beginning readers. Front. Psychol..

[148] Júnior R.d.M., Marinho de Sousa B., Fukusima S. (2014). Hemispheric specialization in face recognition: From spatial frequencies to holistic/analytic cognitive processing. Psychol. Neurosci..

[149] Mayseless N., Breznitz Z. (2011). Brain activity during processing objects and pseudo-objects: Comparison between adult regular and dyslexic readers. Clin. Neurophysiol..

[150] Rüsseler J., Gerth I., Heldmann M., Münte T. (2015). Audiovisual perception of natural speech is impaired in adult dyslexics: An ERP study. Neuroscience.

[151] Rüsseler J., Johannes S., Münte T.F. (2003). Recognition memory for unfamiliar faces does not differ for adult normal and dyslexic readers: An event-related brain potential study. Clin. Neurophysiol..

[152] Tarkiainen A., Helenius P., Salmelin R. (2003). Category-specific occipitotemporal activation during face perception in dyslexic individuals: An MEG study. Neuroimage.

[153] Calder A.J., Young A.W. (2005). Understanding the recognition of facial identity and facial expression. Nat. Rev. Neurosci..

[154] Sinha P., Balas B., Ostrovsky Y., Russell R. (2006). Face recognition by humans: Nineteen results all computer vision researchers should know about. Proc. IEEE.

[155] Collins E., Dundas E., Gabay Y., Plaut D.C., Behrmann M. (2017). Hemispheric organization in disorders of development. Vis. Cogn..

[156] Raman I. (2011). The role of age of acquisition in picture and word naming in dyslexic adults. Br. J. Psychol..

[157] Denckla M.B., Rudel R.G. (1976). Naming of object-drawings by dyslexic and other learning disabled children. Brain Lang..

[158] McGugin R.W., Richler J.J., Herzmann G., Speegle M., Gauthier I. (2012). The Vanderbilt Expertise Test reveals domain-general and domain-specific sex effects in object recognition. Vis. Res..

[159] Huestegge L., Rohrßen J., van Ermingen-Marbach M., Pape-Neumann J., Heim S. (2014). Devil in the details? Developmental dyslexia and visual long-term memory for details. Front. Psychol..

[160] Z. Brachacki G.W., Nicolson R.I., Fawcett A.J. (1995). Impaired recognition of traffic signs in adults with dyslexia. J. Learn. Disabil..

[161] Gabay Y., Dundas E., Plaut D., Behrmann M. (2017). Atypical perceptual processing of faces in developmental dyslexia. Brain Lang..

[162] Sigurdardottir H.M., Fridriksdottir L.E., Gudjonsdottir S., Kristjánsson Á. (2018). Specific problems in visual cognition of dyslexic readers: Face discrimination deficits predict dyslexia over and above discrimination of scrambled faces and novel objects. Cognition.

[163] Holmes D.R., McKeever W.F. (1979). Material specific serial memory deficit in adolescent dyslexics. Cortex.

[164] Liberman I.Y., Mann V.A., Shankweiler D., Werfelman M. (1982). Children’s memory for recurring linguistic and nonlinguistic material in relation to reading ability. Cortex.

[165] Korinth S.P., Sommer W., Breznitz Z. (2012). Does silent reading speed in normal adult readers depend on early visual processes? Evidence from event-related brain potentials. Brain Lang..

[166] Kühn C.D., Gerlach C., Andersen K.B., Poulsen M., Starrfelt R. (2021). Face recognition in developmental dyslexia: Evidence for dissociation between faces and words. Cogn. Neuropsychol..

[167] Benton A.L., Varney N.R., Hamsher K.d. (1978). Visuospatial judgment: A clinical test. Arch. Neurol..

[168] Sigurdardottir H.M., Hjartarson K.H., Gudmundsson G.L., Kristjánsson Á. (2019). Own-race and other-race face recognition problems without visual expertise problems in dyslexic readers. Vis. Res..

[169] Pontius A.A. (1976). Dyslexia and specifically distorted drawings of the face—A new subgroup with prosopagnosia-like signs. Experientia.

[170] Pontius A.A. (1983). Links between literacy skills and accurate spatial relations in representations of the face: Comparison of preschoolers, school children, dyslexics, and mentally retarded. Percept. Mot. Ski..

[171] Aaron P. (1978). Dyslexia, an imbalance in cerebral information-processing strategies. Percept. Mot. Ski..

[172] Duchaine B., Nakayama K. (2006). The Cambridge Face Memory Test: Results for neurologically intact individuals and an investigation of its validity using inverted face stimuli and prosopagnosic participants. Neuropsychologia.

[173] Farah M.J. (2004). Visual Agnosia.

[174] Peterson M.A., Rhodes G. (2003). Perception of Faces, Objects, and Scenes: Analytic and Holistic Processes.

[175] Jozranjbar B., Kristjansson A., Sigurdardottir H.M. (2021). Featural and configural processing of faces and houses in matched dyslexic and typical readers. Neuropsychologia.

[176] Norcia A.M., Appelbaum L.G., Ales J.M., Cottereau B.R., Rossion B. (2015). The steady-state visual evoked potential in vision research: A review. J. Vis..

[177] Lochy A., Van Belle G., Rossion B. (2015). A robust index of lexical representation in the left occipito-temporal cortex as evidenced by EEG responses to fast periodic visual stimulation. Neuropsychologia.

